# Antibiofilm activity of carotenoid crocetin against Staphylococcal strains

**DOI:** 10.3389/fcimb.2024.1404960

**Published:** 2024-05-13

**Authors:** Saurav Paramanya, Jin-Hyung Lee, Jintae Lee

**Affiliations:** School of Chemical Engineering, Yeungnam University, Gyeongsan, Republic of Korea

**Keywords:** antibiofilm, crocetin, fibrils, Staphylococcus aureus, Staphylococcus epidermidis

## Abstract

*Staphylococcus aureus* and *Staphylococcus epidermidis* stand as notorious threats to human beings owing to the myriad of infections they cause. The bacteria readily form biofilms that help in withstanding the effects of antibiotics and the immune system. Intending to combat the biofilm formation and reduce the virulence of the pathogens, we investigated the effects of carotenoids, crocetin, and crocin, on four Staphylococcal strains. Crocetin was found to be the most effective as it diminished the biofilm formation of *S. aureus* ATCC 6538 significantly at 50 µg/mL without exhibiting bactericidal effect (MIC >800 µg/mL) and also inhibited the formation of biofilm by MSSA 25923 and *S. epidermidis* at a concentration as low as 2 µg/mL, and that by methicillin-resistant *S. aureus* MW2 at 100 µg/mL. It displayed minimal to no antibiofilm efficacy on the Gram-negative strains *Escherichia coli* O157:H7 and *Pseudomonas aeruginosa* as well as a fungal strain of *Candida albicans*. It could also curb the formation of fibrils, which partly contributes to the biofilm formation in *S. epidermidis*. Additionally, the ADME analysis of crocetin proclaims how relatively non-toxic the chemical is. Also, crocetin displayed synergistic antibiofilm characteristics in combination with tobramycin. The presence of a polyene chain with carboxylic acid groups at its ends is hypothesized to contribute to the strong antibiofilm characteristics of crocetin. These findings suggest that using apocarotenoids, particularly crocetin might help curb the biofilm formation by *S. aureus* and *S. epidermidis.*

## 1 Introduction

Biofilms are a complex matrix of microorganisms adhering to biotic or abiotic surfaces, typically forming an amalgamation of polysaccharides, proteins, and organic components, prevalent in diverse environments such as clinical and industrial settings, food processing facilities, and drinking water distribution systems (Bazargani and Rohloff, 2016; Khan et al., 2020). Such biofilms pose a grave threat to humans owing to the exacerbated resistance to antimicrobials and host defenses (Gebreyohannes et al., 2019), thus increasing the susceptibility to chronic infections.

*Staphylococcus aureus* is a prevalent human pathogen capable of causing a range of infectious diseases, including skin and soft tissue infections, endocarditis, osteomyelitis, and severe pneumonia leading to fatalities (Guo et al., 2020). The bacterium employs a complex network to orchestrate the synthesis of virulence factors like hemolysins, enterotoxins, and immune-evasive staphyloxanthin. These factors contribute to various life-threatening infections such as bacteremia, pulmonary infections, gastroenteritis, and toxic shock syndrome (Valliammai et al., 2020; Park et al., 2023). The strain’s propensity to form biofilms on damaged tissues and medical implants contributes to its development of antibiotic resistance and virulence (Moormeier and Bayles, 2017). Since the introduction of penicillin in the 1940s and methicillin in the 1950s to the present (Nandhini et al., 2022), *Staphylococcus aureus* has adapted rapidly by becoming resistant to each new type of drug. The ability to combat the effect of drugs led it to become the main cause of community-acquired and nosocomial infections resulting in high morbidity and mortality rates (Tong et al., 2015).

*Staphylococcus epidermidis* is the most frequently encountered Coagulase-negative staphylococci (CoNS) implicated in clinical diseases (Argemi et al., 2019).

CoNS can lead to severe infections affecting various areas such as the bloodstream, skin, soft tissues, urinary tract, prosthetic joints, and vascular grafts, with *S. epidermidis* particularly implicated in catheter-related urinary tract infections (Michels et al., 2021; Rubini et al., 2021). Prolonged intensive care practices have led to a quintessential commensal organism like *S. epidermidis* inducing a multitude of infections by evading the host’s defense mechanisms and developing resistance to multiple drugs (Kleinschmidt et al., 2015).

A class of isoprenoid derivatives called carotenoids are biosynthesized by microorganisms, fungi, algae, and plants which serve as effective free-radical scavengers (Mohd Hatta et al., 2023). These carotenoids find their way into animals through fruits and vegetables wherein they undergo oxidative cleavage for the retinal formation and activation of the transcriptional system. The cleavage process results in products, including apocarotenoids, recognized for their biological activity as anticancer agents and cell modulators (Kim et al., 2014). One such apocarotenoid, crocetin, found in saffron, is renowned for its wide range of biological activities in addition to its high therapeutic index (Gupta, 2019). Although saffron possesses a plethora of medicinal benefits owing to the presence of active ingredients and found enormous usage in ancient times, there are no studies regarding the antibiofilm characteristics of crocetin or the other active ingredients (Carradori et al., 2016; Butnariu et al., 2022). The anticancerous property of crocetin has been studied extensively (Colapietro et al., 2019), but there was a need for its antibiofilm property to be assessed to help combat the drug resistance by the pathogens.

The following study was conducted to find a compound that could hinder the formation of biofilm and toxin production by *S. aureus* and *S. epidermidis* without decimating the bacterium. The two most active ingredients of saffron, crocetin and crocin were used to assess their antibiofilm attributes against *S. aureus* and *S. epidermidis*. Crocetin and crocin share structural resemblances with fatty acids. Similar to fatty acids, crocetin possesses a long alkyl chain terminated by carboxylic acid groups at its ends, while crocin differs in its end groups, containing gentiobiose instead. Fatty acids are recognized as established antibiofilm agents, effectively curbing the growth and formation of biofilms across various pathogens (Kumar et al., 2020). Due to the high resemblance of crocetin to fatty acids and its superior activity compared to crocin, it was taken into account for the further investigation of its activity against the other four *Staphylococcus* strains, *Staphylococcus aureus* ATCC 6538, *Staphylococcus aureus* ATCC 25923, *Staphylococcus epidermidis* ATCC 14990, and a methicillin-resistant *Staphylococcus aureus* (MRSA MW2). Live imaging microscopy, scanning electron microscopy, lipase activity, hemolysis, EPS production, metabolic activity, and hydrophobicity assays were committed to evaluate the biofilm-inhibiting and toxin-inhibiting activity of crocetin against the aforementioned strains. Furthermore, ADME analysis was simulated to analyze the toxicity of crocetin and crocin.

## 2 Materials and methods

### 2.1 Bacterial strains, culture media, chemicals, and growth analysis

The strains used for the experiments were two methicillin-sensitive *S. aureus* strains (ATCC 6538 and ATCC 25923), a methicillin-resistant *S. aureus* strain (MW2), an *S. epidermidis* strain (ATCC 14990), a fungal strain of *C. albicans* DAY185, an enterohaemorrhagic *E. coli* O157:H7 strain, and a *P. aeruginosa* PAO1 strain. Colonies of MSSA ATCC 6538, MSSA ATCC 25923, *S. epidermidis* ATCC 14990, *E. coli* O157:H7, and *P. aeruginosa* PAO1 were inoculated in Luria-Bertani (LB) medium at 37 °C while MRSA MW2 was cultivated in LB medium containing 0.2% of glucose under the same condition. The fungal strain, *C. albicans* DAY185 was inoculated in Potato Dextrose Broth (PDB) at 37 °C. *V. parahaemolyticus* was cultured in mLB (mineral Luria-Bertani) medium viz. LB medium supplemented with 3% (w/v) NaCl. The test compounds, crocetin, crocin, and tobramycin along with crystal violet were procured from Sigma-Aldrich (St Louis, MO, USA). Crocetin and crocin were dissolved in Dimethyl Sulfoxide (DMSO) while tobramycin was dissolved in distilled water, and 0.1% of DMSO was used as control as it doesn’t serve as a suitable medium for bacterial growth and biofilm formation. The growth of the bacterium was assessed by forming Colony Forming Units (CFU) on 90 mm petri plates after incubating the MSSA ATCC 6538 in LB medium with or without crocetin or crocin or a combination of tobramycin and crocetin or crocin at 37 °C for 24 hrs.

### 2.2 Crystal-violet biofilm assay

The test compounds’ effect on the biofilm formation was assessed by a crystal-violet biofilm assay as previously reported (Jeon et al., 2024). The cells of *S. aureus* and *S. epidermidis* (~10^7^ CFU/mL) were inoculated in LB medium and crocetin or crocin were added at 0, 5, 10, 20, 50, and 100 µg/mL to the wells of the 96-well plates. To assess the effect of combination of tobramycin with either crocetin or crocin, a crystal violet assay was performed following the methodology reported previously (Park et al., 2022) wherein *S. aureus* cells (~10^7^ CFU/mL) were inoculated in LB medium, and a fixed concentration of tobramycin at 2 µg/mL was added to 96-well plates, along with varying concentrations of crocetin or crocin (0, 20, and 50 µg/mL). The plates were then incubated at 37°C for 24 hrs without agitation. The planktonic cells were discarded by washing the plates well with distilled water three times. The biofilm cells were stained with 0.1% (w/v) of crystal violet for 20 mins. and then washed using distilled water thrice. The stained biofilm cells were dissolved using 95% ethanol with vigorous shaking. The absorbances were read at 570 nm using a Multiskan plate reader (Thermo Fisher Scientific, Waltham, MA, USA). The results of biofilm formation were derived from three independent cultures, each having six replicate wells.

### 2.3 Dispersal assay

The biofilm dispersal assay was conducted to evaluate how crocetin affects *S. aureus* and *S. epidermidis* using the method previously reported (Sathiyamoorthi et al., 2024). After allowing biofilms to form for 24 hrs at 37°C and the above biofilm assay in the section 2.2 was performed.

### 2.4 Microscopic observations of biofilms

The *S. aureus* and *S. epidermidis* biofilms were formed in 96-well plates as discussed earlier (2.2) in the presence or absence of crocetin (0, 5, 10, 20, 50, and 100 µg/mL) at 37 °C for 24 hrs and the plates were washed thrice with distilled water to discard the planktonic cells followed by staining with 0.1% (w/v) crystal violet and subsequent washing of the stain. The stained biofilms were observed under the iRiS™ Digital Cell Imaging System (Logos Biosystems, Anyang, Korea). ImageJ (https://imagej.nih.gov/ij/index.html) was then employed to generate color-coded 3D biofilm images.

Additionally, the biofilm reduction by crocetin was observed by SEM, as previously reported. *S. aureus* ATCC 6538 and *S. epidermidis* cells (~10^7^ CFU/mL) were inoculated in fresh LB medium with or without crocetin (0, 5, 10, 20, 50, and 100 μg/mL) in a 96-well plate. Each well was equipped with a piece of nylon membrane (~ 0.16 cm^2^) and subsequently, the cells were incubated for 24 h at 37°C without agitation. Biofilms established on the membranes were then fixed with a 1:1 concoction of glutaraldehyde (2.5%) and formaldehyde (2%) for 24 hrs, followed by dehydration with ethanol (50, 70, 90, 95, and 99%) sequentially. Utilizing a critical point dryer (HCP-2, Hitachi, Tokyo, Japan), the nylon membranes were subjected to drying and was proceeded by the metal coating using Precision Etching Coating System (Gatan, Inc., Pleasanton, USA) with subsequent observation using S-4800 (Hitachi, Tokyo, Japan), a field emission scanning electron microscope.

### 2.5 Hemolytic activity assay

A hemolytic activity assay was carried out using sheep blood cells (MB Cell, South Korea) as described previously (Kim et al., 2018). The cells of *S. aureus* (~2×10^7^ CFU/mL) were suspended in 2 mL of LB medium and were cultivated using 0, 20, and 50 µg/mL of crocetin with vigorous shaking at 250 rpm for 24 hrs. Fresh sheep blood was subjected to centrifugation at 3,000 × g for 2 mins., and the red blood cells were collected and then cleaned three times with PBS followed by their resuspension in PBS buffer (3.3%). The cell culture of *S. aureus* (600 µL) was then added to 2 mL of red blood cells and the sample was subjected to 1 hour of shaking at 250 rpm at 37 °C. The mixtures underwent centrifugation for 10 mins. at 16,600 × g, and the absorbances of supernatants were gauged at 543 nm.

### 2.6 Exopolysaccharide production assay

An assay was performed to estimate the production of exopolysaccharides as previously described (Ahmed et al., 2022). Briefly, *S. epidermidis* was incubated with or without crocetin at 5, 10, and 20 μg/mL in LB with shaking at 250 rpm for 24 h at 37°C. The tubes underwent centrifugation at 10,000 rpm for 10 mins. and the resulting supernatants were combined with chilled ethanol in a ratio of 1:3 and allowed to stand undisturbed at 4°C overnight. The suspension was centrifuged at 10,000 rpm for 5 mins. to collect the EPS precipitates followed by subsequent solubilizing in 200 µL of water. A phenol-sulfuric acid mixture was prepared in the ratio of 1:5 and added to the 200 µL samples, followed by incubation at room temperature for 30 minutes, and then left undisturbed for an additional 20 minutes before measuring the absorbances at 490 nm.

### 2.7 Biofilm cell viability assay

The metabolic viabilities of the cells in the biofilms of *S. epidermidis* was estimated using Triphenyl tetrazolium chloride (TTC) assay as described earlier (Faleye et al., 2023) with slight modifications. Briefly, cells of *S. epidermidis* were grown in LB medium overnight and the 1:100 dilution of the same was treated with crocetin at 0, 2, 5, 10, and 20 µg/mL in a 96-well plate. TTC dye was introduced into each well to achieve a final concentration of 0.05% w/v, followed by incubation of the plate at 37°C for 24 hrs. The plate was washed with distilled water to remove the non-adherent cells, and the formazan production by TTC was solubilized in methanol. The absorbance of each well was measured at 500 nm using a Multiskan EX microplate reader. The metabolic viabilities of biofilm cells were quantified and expressed as a percentage of absorbance relative to untreated controls.

### 2.8 Lipase production assay

The effect of crocetin on *S. aureus* was quantified by an extracellular lipase production assay wherein the cells of *S. aureus* (~2×10^7^ CFU/mL) were incubated in 2 mL of LB medium at 37 °C for 20 hrs with constant shaking in the absence or presence of crocetin (0, 20, and 50 µg/mL) as reported earlier (Kim et al., 2022). To summarize, culture supernatants (0.1 mL) were homogenized with 0.9 mL of substrate solution which comprised of 10% of buffer A containing 3 mg/mL of p-nitrophenyl palmitate in isopropyl alcohol and 90% of buffer B containing 1 mg/mL of gum arabic and 2 mg/mL sodium deoxycholate in 50 mM Na_2_PO_4_ buffer, followed by heating at 40°C for 30 mins. 1 M Na_2_CO_3_ was added to cease the reactions and the reaction supernatants’ absorbances were quantified at 405 nm.

### 2.9 Hydrophobicity assay

A hydrophobicity assay was carried out as described previously (Lee et al., 2016). *S. aureus* and *S. epidermidis* cells were incubated overnight in LB medium at 37 °C and were garnered after centrifuging them at 7,000×g for 5 mins. The garnered cells were mixed with 2 mL of PBS and 300 µL of hexadecane followed by a vortexing of 4 mins. The sample containing tubes was allowed to rest for 30 mins. for the phases to separate. The aqueous phases were used to measure the absorbances at OD_600_. Cell surface hydrophobicity (H%) was expressed by measuring the reduction in optical density (OD) of the aqueous phase and calculated using the equation H% = [(ODo - OD)/ODo] × 100, where ODo represents the initial OD before extraction with hexadecane, and OD represents the OD after extraction.

### 2.10 Evaluation of absorption, distribution, metabolism, and excretion properties

ADME software was accessed to appraise the drug-like properties of crocetin and crocin. The online web servers (https://preadmet.qsarhub.com/), (https://www.molinspiration.com), and GUSAR (https://www.way2drug.com/) were accessed on 10^th^ November, 2023.

### 2.11 Statistical analysis

The statistical analysis was conducted using one-way ANOVA followed by Dunnett’s test in SPSS version 23 (SPSS Inc., Chicago, IL, USA). Mean values accompanied by standard deviations were reported, with significance considered at p-values below 0.05.

## 3 Results

### 3.1 Antibiofilm activities of the apocarotenoids against *S. aureus* and *S. epidermidis*

**Figure 1:**
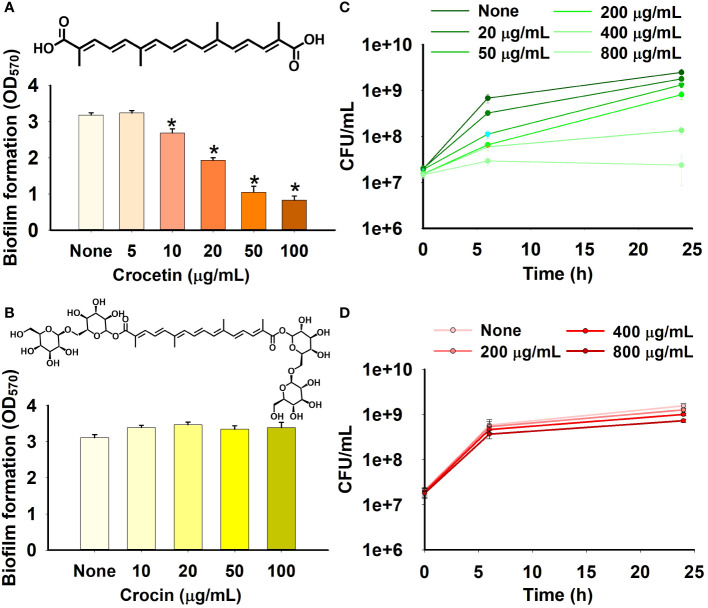
Effect of crocetin and crocin on biofilm formation and cell growth. Biofilm formation by *S. aureus* ATCC 6538 in the presence of crocetin **(A)**, crocin **(B)**. *S. aureus* cell growth in the presence of crocetin **(C)**, and crocin **(D)**. *p<0.05 *vs* untreated controls (None).

**Figure 2:**
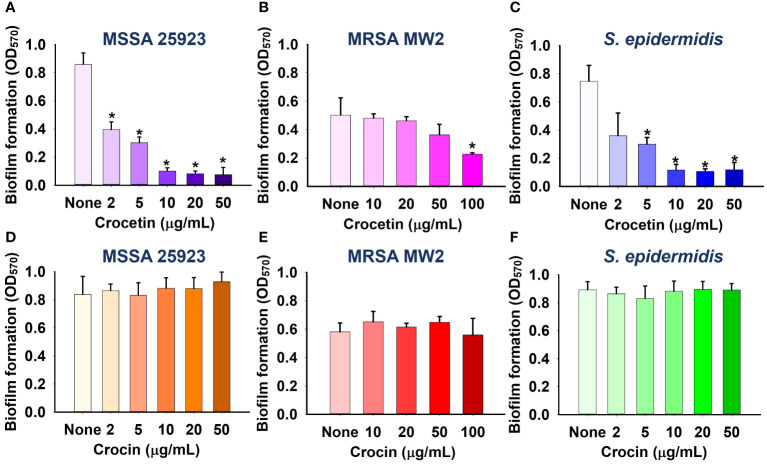
Effect of crocetin on biofilm formation by *S. aureus* ATCC 25923 **(A)**, MRSA MW2 **(B)**, and *S. epidermidis* **(C)**, and effect of crocin on biofilm formation by *S. aureus* ATCC 25923 **(D)**, MRSA MW2 **(E)**, and *S. epidermidis* **(F)**. *p<0.05 *vs* untreated controls (None).

The apocarotenoids, namely, crocetin and crocin were tested at concentrations up to 100 µg/mL for *S. aureus* ATCC 6538 and MRSA MW2, and 50 µg/mL for *S. aureus* ATCC 25923 and *S. epidermidis* to assess their antibiofilm efficiencies against the aforementioned pathogens. Only crocetin showed a significant effect on the biofilm formation while crocin remained ineffective (**Figures 1B**, **2D–F**). Crocetin could inhibit the biofilm growth by *S. epidermidis* and *S. aureus* ATCC 25923 by 52% and 55% respectively at a concentration of 2 µg/mL (**Figures 2A, C**) while in the case of *S. aureus* ATCC 6538, a reduction of 67% was observed at a higher concentration of 50 µg/mL (**Figure 1A**). A reduction of 55% in biofilm formation was observed in MRSA MW2 at a high concentration of 100 µg/mL (**Figure 2B**). The effect of crocetin on the cell growth of *S. aureus* ATCC 6538 was observed by performing colony forming unit (CFU) assay wherein a delay in the growth of planktonic cells was observed starting from the lowest tested concentration, 20 µg/mL until 400 µg/mL; however, at 800 µg/mL, the compound started exhibiting bacteriostatic effect (**Figure 1C**). CFU assay of the same bacterium using crocin showed no significant effect on the cell growth of the pathogen even at high concentrations (**Figure 1D**). These results suggest that crocetin dwindles the biofilm formation without exhibiting a significant effect on the planktonic cell growth. On the other hand, the dispersal assay showed that mature biofilms of *S. aureus* and *S. epidermidis* were not affected when treated with crocetin at concentrations of 20 and 50 µg/mL (**Supplementary Figures 3A, B**), which confirmed that biofilm dispersal is much difficult than biofilm inhibition.

### 3.2 Antibiofilm characteristics of crocetin on other microbes

**Figure 3:**
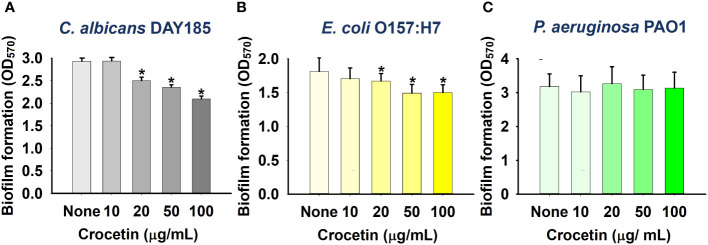
Effect of crocetin on biofilm formation by *C. albicans* DAY185 **(A)**, *E*. *coli* O157:H7 **(B)**, and *P. aeruginosa* PAO1 **(C)**. *p<0.05 *vs* untreated controls (None).

The biofilm assay was further performed on other pathogens, namely, *C. albicans* DAY185, a fungal strain, as well as two Gram-negative strains: *E. coli* O157:H7 and *P. aeruginosa* PAO1. While the fungal strain (*C. albicans* DAY185) and Gram-negative strain of *E. coli* O157:H7 were scarcely affected at the same concentrations (**Figures 3A, B**), crocetin, unlike the other pathogens listed above, did not affect *P. aeruginosa* PAO1 biofilm development (**Figure 3C**).

### 3.3 Synergistic antibiofilm characteristics of the combination of tobramycin with crocetin

**Figure 4:**
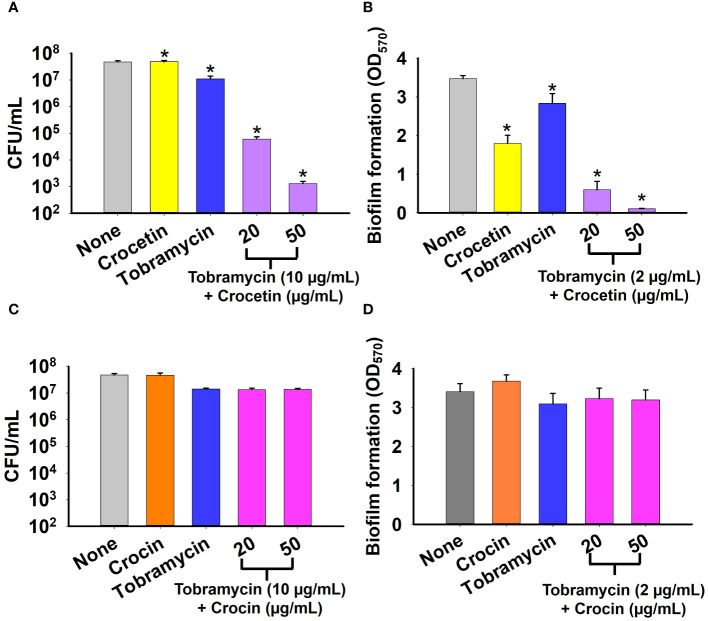
Effect of combination of tobramycin with crocetin or crocin on cell survival and biofilm formation. *S. aureus* cell survival in the presence of tobramycin and crocetin **(A)**, and tobramycin and crocin **(C)**. Biofilm formation by *S. aureus* ATCC 6538 in the presence of tobramycin and crocetin **(B)**, and tobramycin and crocin **(D)**. *p<0.05 *vs* untreated controls (None).

The biofilm assay was conducted on *S. aureus* to evaluate the effect of combining tobramycin with either crocetin or crocin. The CFU results demonstrate a slight reduction of less than 10-fold in the colonies formed by *S. aureus* when treated with tobramycin alone, with no significant change observed in the presence of crocetin alone. Combining 10 µg/mL of tobramycin with 20 µg/mL of crocetin resulted in approximately a 1000-fold reduction, while the reduction increased to around 10,000-fold with 50 µg/mL of crocetin and the same concentration of tobramycin (**Figure 4A**). The biofilm assay revealed that tobramycin alone at a concentration of 2 µg/mL reduced biofilm growth by 19%, while crocetin alone at 20 µg/mL decreased it by 48%. However, when crocetin was combined with 2 µg/mL of tobramycin at concentrations of 20 and 50 µg/mL, the reduction in biofilm growth was notably higher at 83% and 97%, respectively. (**Figure 4B**). Nonetheless, the combination of tobramycin with crocin did not impact CFU or biofilm growth by *S. aureus* (**Figures 4C, D**).

### 3.4 Microscopic findings of crocetin’s effect on *S. aureus* and *S. epidermidis*

**Figure 5:**
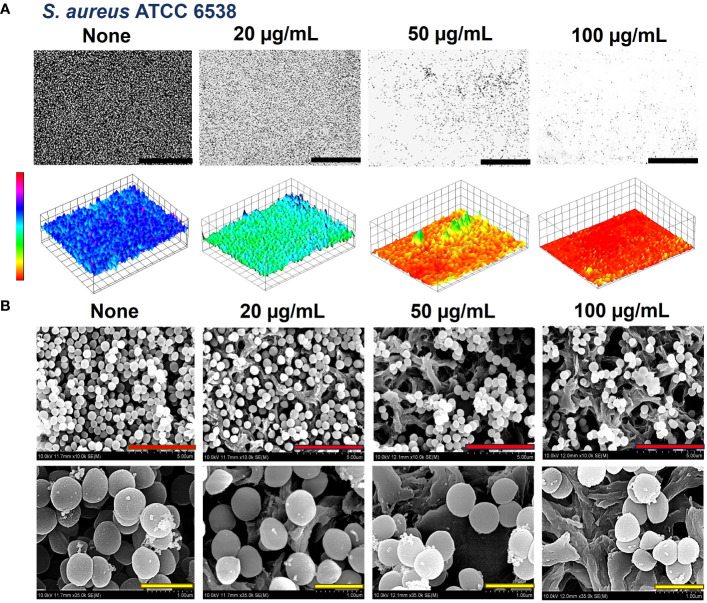
Antibiofilm effect of crocetin on *S. aureus*. Constructed color-coded 3D images of *S. aureus* ATCC 6538 in the presence of crocetin **(A)** and corresponding SEM images **(B)**. 50, 5, and 1 µm are represented by black, red, and yellow scale bars respectively.

**Figure 6:**
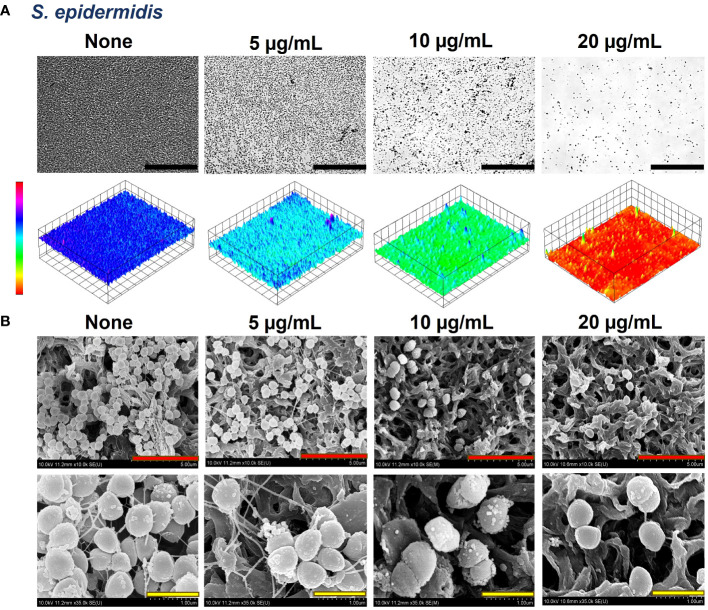
Antibiofilm effects of crocetin on *S. epidermidis*. Constructed color-coded 3D images of *S. epidermidis* in the presence of crocetin **(A)** and corresponding SEM images **(B)**. 50, 5, and 1 µm are represented by black, red, and yellow scale bars respectively. .

The biofilms formed by the bacteria were observed by live-imaging microscopy and SEM. 3D color images that were obtained through live-imaging microscopy showed blue or bluish-cyan for ‘None’ indicating strong high biofilms (**Figures 5A**, **6A**, **Supplementary Figure 1**). At concentrations of 50 and 100 µg/mL (for *S. aureus* ATCC 6538) and 10 and 20 µg/mL (*S. aureus* ATCC 25923 and *S. epidermidis*), red color with patches of yellow exhibited scarcely to no biofilm formation owing to crocetin’s antibiofilm attributes (**Figures 5A**, **6A**, **Supplementary Figure 1**). The SEM images were found to be following the results of the biofilm assay. The cell growth could be seen decreasing in a dose-dependent manner owing to the decrease in biofilm formation without imparting any changes to the morphology of the bacterial cells (**Figures 5B**, **6B**). Furthermore, fibril production by *S. epidermidis* decreased in a dose-dependent manner, with untreated sample demonstrating significant fibrils in their respective images. The reduction in fibrils, which play a role in the formation of biofilms in *S. epidermidis*, could be correlated with a significant reduction in its biofilms. Presumably, crocetin curtails biofilm development by targeting the fibril growth in *S. epidermidis*. This concludes that crocetin is capable of inhibiting the biofilm formation of the bacteria in question without affecting their cell morphology.

### 3.5 Effect of crocetin on *S. aureus’* hemolytic activity, hydrophobicity, and lipase production

A myriad of virulence factors is produced by *S. aureus* which includes hemolysin, and extracellular lipase which called for the need to investigate the effect of crocetin on them. Crocetin up to 50 µg/mL showed ineffectiveness in hindering lipase production by the bacterium (**Supplementary Figure 2A**), preventing hemolysis of sheep red blood cells (RBCs) (**Supplementary Figure 2B**), and did not enhance bacterial affinity towards the aqueous phase in the hydrophobicity assay against *S. aureus* (**Supplementary Figure 2C**).

### 3.6 Effect of crocetin on *S epidermidis’* EPS production, metabolic activity, and hydrophobicity

Crocetin up to 20 µg/mL was found ineffective in preventing EPS production by *S. epidermidis* cells (**Supplementary Figure 2D**), had no impact on the metabolic activity of biofilm cells (**Supplementary Figure 2E**), and did not alter the bacterium’s hydrophobicity (**Supplementary Figure 2F**).

### 3.7 Crocetin and crocin ADME profiling

Crocetin and crocin ADME profiles found that the former violated one and the latter three of Lipinski’s five rules, with both compounds having miLogP<5. The skin permeability of both substances was adequate, but they differed significantly in terms of human intestine absorption, which was high for crocetin (>95%), but severely low for crocin (1%). Crocetin was discovered to have no fish toxicity, however, crocin demonstrated a high toxicity for the same. Both compounds were found to induce carcinogenic properties in mice. The molecular volume, a determining factor for the transport characteristics of compounds, was found to be higher in crocin compared to crocetin. This difference could elucidate the superior absorption of crocetin over crocin, which eventually metabolizes into crocetin and glucuronide conjugates. Another significant factor influencing transport characteristics, the Total Polar Surface Area (TPSA), was found to exceed the ideal score of 140 Å in the case of crocin, while it was almost half the ideal score in the case of crocetin. This further attests the superior absorptivity of crocetin compared to crocin. The observation that the two compounds in question are environmentally non-toxic suggests their potential as green alternatives. **Table 1** contains an in-depth breakdown of ADME parameters.

**Table 1:** ADME (absorption, distribution, metabolism, and excretion) profiles of crocetin and crocin.

Property	Crocetin	Crocin
Lipinski rule of five	Violated	Violated
Lead like violations	1	2
Lipinski rule of five violations	0	3
Plasma protein binding	94.835851	26.980257
Blood brain barrier permeability	1.08903	0.0274059
Skin permeability	-0.954783	-2.32731
Human intestinal absorption	96.707985	0.164266
Caco2	21.1005	12.2488
Mouse carcinogenicity	Positive	Positive
Acute fish toxicity (medaka)	0.00111522	78.6123
Acute fish toxicity (minnow)	0.00155463	96.7506
*In vitro* hERG inhibition	Medium risk	Ambiguous
miLogP	4.63	-1.45
Mol volume	324.80	861.10
TPSA	74.60	381.97
GPCR ligand	0.13	-3.49
Ion channel modulator	0.19	-3.67
Kinase inhibitor	-0.01	-3.66
Nuclear receptor ligand	0.68	-3.62
Protein inhibitor	-0.04	-3.18
Enzyme inhibitor	0.40	-3.48
Rat IP LD50 classification	Nontoxic in AD	Nontoxic in AD
Rat IV LD50 classification	Class 5 in AD	Nontoxic in AD
Rat oral LD50 classification	Nontoxic in AD	Class 5 out of AD
Rat SC LD50 classification	Class 5 in AD	Nontoxic in AD

## 4 Discussion

This study reveals that the saffron-derived crocetin exerts substantial antibiofilm effects on *S. aureus* and *S. epidermidis* strains without exhibiting any bactericidal effects. The non-toxicity of crocetin along with crocin has also been established by the ADME profiles of the same.

Saffron has widely been used as an effective treatment in ancient India and China and is known to possess a plethora of active components including crocetin and crocin, and extensive research has suggested its potential efficacy in addressing various health conditions, including diabetes mellitus, cancers, Alzheimer’s disease, and numerous other ailments (Razak et al., 2017). *S. aureus* biofilms have been associated with chronic wound infections as well as a common skin disorder, acne vulgaris, which is primarily caused by *P. acnes* but exacerbated by the presence of *S. aureus* biofilms (Archer et al., 2011; Tyner and Patel, 2016). Thus, the inhibitory effect of crocetin on *S. aureus* biofilms holds promise in aiding the treatment of such conditions.

Our research highlights that crocetin, our test compound, notably reduces biofilm formation only in Gram-positive strains, showing minimal to no effect on Gram-negative bacteria and the fungal strain, *Candida albicans* (**Figures 1**–**3**). Gram-negative bacteria possess an outer membrane that sets them apart from Gram-positive bacteria, rendering them more resistant to antibiotics (Bann et al., 2021). While this membrane permits the passage of smaller molecules, bacteria can modify it by altering its hydrophobic properties or through mutations in porins, conferring resistance to several antibiotics, including β-lactams (Breijyeh et al., 2020). Thus, enhancing compound penetration through this outer membrane is crucial for antibiotic efficacy (Miller, 2016). It is hypothesized that crocetin’s limited penetration through the outer membrane of Gram-negative bacteria accounts for its reduced effectiveness against them. Similarly, the unique composition of fungal cell membranes compared to bacterial ones requires specific antifungal binding to fungal membrane components for optimal penetration and efficacy (Osset-Trénor et al., 2023). Antifungals typically act by creating pores in the fungal membrane to facilitate the passage of smaller molecules or by targeting genes and enzymes essential for membrane function (Costa-de-Oliveira and Rodrigues, 2020). Crocetin might not achieve these actions at a sufficient level, explaining its diminished effectiveness against fungi.

Our study demonstrates the presence of fibrils dispersed throughout the biofilms of *S. epidermidis*, which are recognized contributors to the pathogen’s biofilm-forming ability (**Figure 6**). In the mature biofilms of *S. epidermidis*, amyloid fibrils are formed by accumulated-associated protein (aap) and small basic protein (sbp). The A domain of the aap facilitates adhesion to unconditioned biomaterial, while sbp contributes to the integrity of both protein and polysaccharide biofilm matrices (Foster, 2020). There are reports indicating the presence of fibrils as localized tufts on *S. epidermidis* cells, which impact the cell surface hydrophobicity and, to some extent, the biofilm-forming ability, although not significantly (Banner et al., 2007). Another study involving the same strain suggests that Aap plays a role in skin colonization by mediating adhesion to corneocytes (Macintosh et al., 2009). A recent study indicates that Sbp has the ability to form functional amyloid fibrils, elucidating its function as a scaffolding protein in *S. epidermidis* biofilm formation (Wang et al., 2018). Interestingly, the biofilms of *S. aureus* did not display the presence of such fibrils (**Figure 5**). It’s hypothesized that crocetin encumbers the biofilm development of the tested strains in the same manner. We suggest the elucidation of the upregulation and downregulation patterns of genes associated with biofilm formation in both *S. aureus* and *S. epidermidis*. The mounting evidence indicates that changes in gene expression during initial bacterial adherence and intercellular adhesion (biofilm formation) offer propitious avenues for novel therapeutic interventions targeting *S. aureus* and *S. epidermidis* biofilm formation, presenting potentially advantageous alternatives to existing treatments.

Carotenoids are characterized by their chemical structure, which in turn dictates their potential biological functions. They are capable of absorbing surplus energy from the other molecules owing to their polyene backbone having a distinct arrangement of single and double bonds while the particular end groups can affect their polarity (Zeb and Mehmood, 2004).

**Figure 7:**
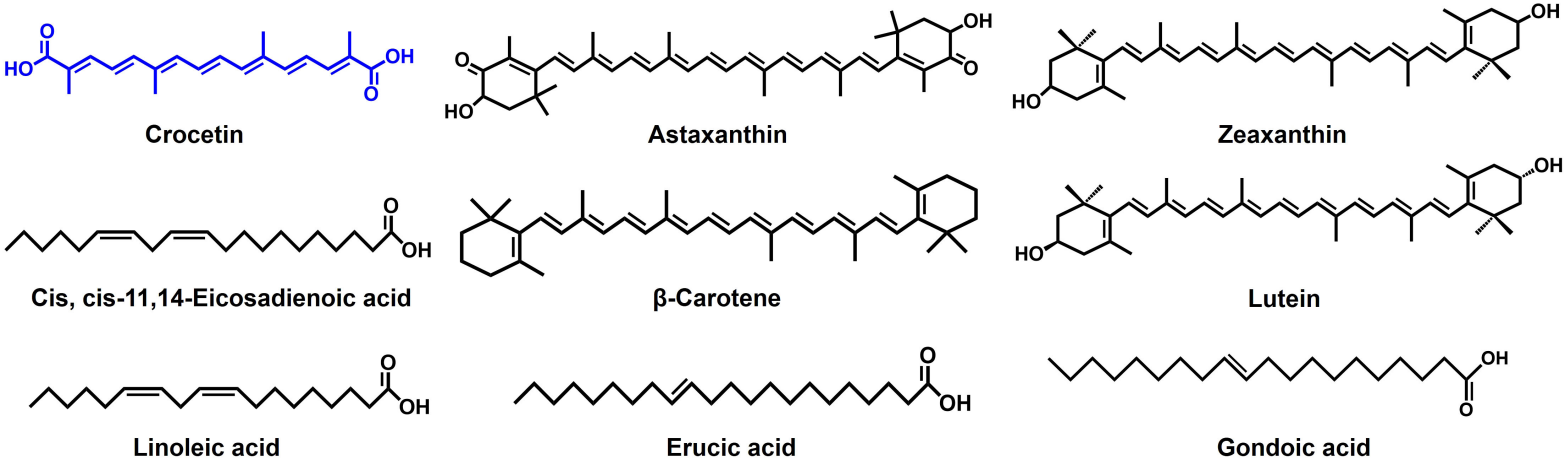
Structures of compounds reported to have antibiofilm activity.

Numerous phytochemicals, including carotenoids, have been reported to demonstrate antibiofilm and antimicrobial properties (Kim et al., 2024). Astaxanthin, an algae-derived carotenoid has been reported to dwindle the biofilm growth in *S. aureus* by mitigating its adherence to the surface and in *S. epidermidis* by expressing a bactericidal effect (Weintraub et al., 2017; Haasbroek et al., 2022). Additionally, a research investigation explores the antibiofilm characteristic of zeaxanthin, which disrupts the quorum sensing (QS) systems and inhibits biofilm formation in *P. aeruginosa* (Gökalsın et al., 2017). A related investigation involving lutein on the identical strain highlights the compound’s capacity to impede biofilm formation by degrading extracellular polymeric substances (EPS), which provide stability to the biofilms (Sampathkumar et al., 2019). The structural resemblances of these carotenoids to crocetin (**Figure 7**) suggest that the polyene backbone could play a role in influencing their antibiofilm properties.

Crocetin, categorized as a lipophilic carotenoid, contrasts with crocin, which serves as the hydrophilic diester of crocetin combined with gentiobiose (Zhao et al., 2021). While crocetin demonstrated robust antibiofilm activity against staphylococcal strains and exhibited antibacterial effects at relatively high concentrations, crocin, on the other hand, proved ineffective in displaying antibiofilm or antibacterial properties (**Figures 1**, **2**). It’s intriguing that despite sharing the polyene backbone characteristic with crocetin and other carotenoids discussed above (**Figures 1B**, **7**), crocin was incompetent in controlling the biofilm formation and growth. This is an implication of the end groups playing an equally consequential role in controlling the antibiofilm characteristics of the carotenoids. Perhaps, the gentiobiose substitution in crocetin to form crocin denigrates its antibiofilm property. The unbound carboxylic acid groups in crocetin promote an increase in its acidity and stabilization through conjugation, unlike crocin, where the gentiobiose residues do not participate in conjugation (Akhtari et al., 2013). This attribute might potentially enhance the antibiofilm properties of crocetin and analogous compounds. It’s been previously reported that the combination of tobramycin with myristoleic acid effectively suppresses the CFU count and inhibits biofilm formation by *S. aureus* (Park et al., 2022). Our research findings demonstrate analogous outcomes when tobramycin is employed concurrently with crocetin on the same strain. Fatty acids, particularly at high concentrations, exhibit antimicrobial activities by potentially altering cell membrane permeability, disrupting membranes, and causing leakage of intracellular metabolites, while another study found that the combined antibiofilm effect of lauric acid with gentamicin and streptomycin was linked to its surfactant properties, aiding in the penetration of the biofilm matrix (Desbois and Smith, 2010; Hess et al., 2014). Because crocetin shares structural similarities with fatty acids, we hypothesize that it works on *S. aureus* in a manner similar to that of fatty acids. However, the mechanism by which crocetin curtails the formation and growth of staphylococcal biofilms remains ambiguous, underscoring the necessity to comprehend its interaction with bacterial cells and the biofilm matrix. Thus, we suggest delineating the mechanism of action of crocetin on the biofilms of *S. aureus* and *S. epidermidis* which can provide a better understanding of the efficacy of the compound in question.

The oral intake of crocetin has been linked to its swift absorption into the bloodstream in the case of both mice and humans (Asai et al., 2005; Umigai et al., 2011). In mice, crocetin persists either as an intact free form or as its glucuronide conjugates, while crocin, following oral administration, hydrolyzes into crocetin either before or after intestinal absorption (Zhang et al., 2017).

The compounds under investigation demonstrated antidepressant effects in mice, with crocetin proving to be more effective than crocin (Amin et al., 2015). Following its daily oral administration, crocetin has been observed to improve physical performance in humans, particularly among men, during tests that induce fatigue (Mizuma et al., 2009). These findings suggest that orally incorporating crocetin offers multiple benefits and may also serve as a promising mode of administration for treating biofilm-associated infections caused by *S. aureus* and *S. epidermidis*. Moreover, our ADME results (**Table 1**) showcase that crocetin is environmentally innocuous and exhibits substantial skin permeability, attesting its potential for dermal administration in treating topical biofilm-associated infections caused by the mentioned strains.

Crocetin was identified as a potent antibiofilm agent against the tested strains and its inhibitory potential was found to be enhanced through synergistic administration with an aminoglycoside for treating staphylococcal infections. When utilized alongside a low dosage of tobramycin, it has the potential to markedly reduce both colony formation and biofilm development by *S. aureus*, rendering it a promising candidate for inclusion in treatment regimens (**Figures 4A, B**). While the mechanism of action remains unclear, this aspect has the potential to revolutionize the medical field, especially considering the significant challenges posed by antimicrobial resistance.

## Data availability statement

The original contributions presented in the study are included in the article/**Supplementary Material**. Further inquiries can be directed to the corresponding author.

## Author contributions

SP: Investigation, Methodology, Software, Validation, Writing – original draft, Writing – review & editing. J-HL: Conceptualization, Funding acquisition, Methodology, Project administration, Resources, Supervision, Validation, Writing – original draft, Writing – review & editing. JL: Conceptualization, Funding acquisition, Project administration, Writing – original draft, Writing – review & editing.
