# Supplementary material for: Antibiofilm activity of carotenoid crocetin against Staphylococcal strains
Source: Front Cell Infect Microbiol. 2024 May 13;14:1404960. doi: 10.3389/fcimb.2024.1404960 (PMC11128560; doi:10.3389/fcimb.2024.1404960)
Supplement: Supplementary file 1 [file DataSheet_1.docx]

**Supplementary information**

**Antibiofilm activity of carotenoid crocetin against Staphylococcal strains**

Saurav Paramanya, Jin-Hyung Lee, and Jintae Lee*

School of Chemical Engineering, Yeungnam University, Gyeongsan, Republic of Korea

*Correspondence: Jintae Lee, [jtlee@ynu.ac.kr](mailto:jtlee@ynu.ac.kr)

Running title: Antibiofilm activity of crocetin


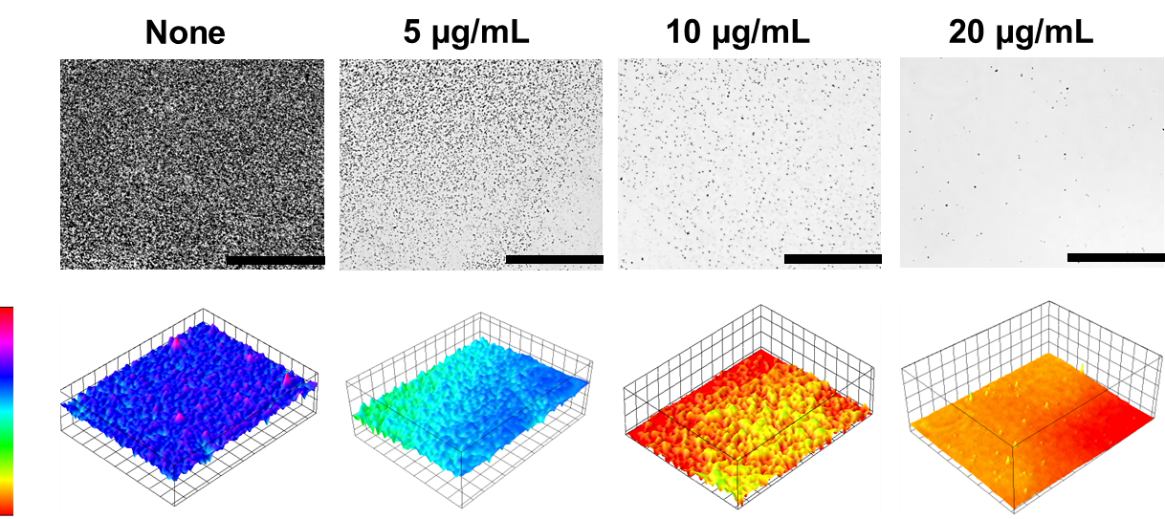


**Supplementary Figure 1.** Antibiofilm effects of crocetin on *S. aureus* ATCC 25923. Constructed colour-coded 3D images of *S. aureus* ATCC 25923 in the presence of crocetin. Black scale bars represent 50 µm.


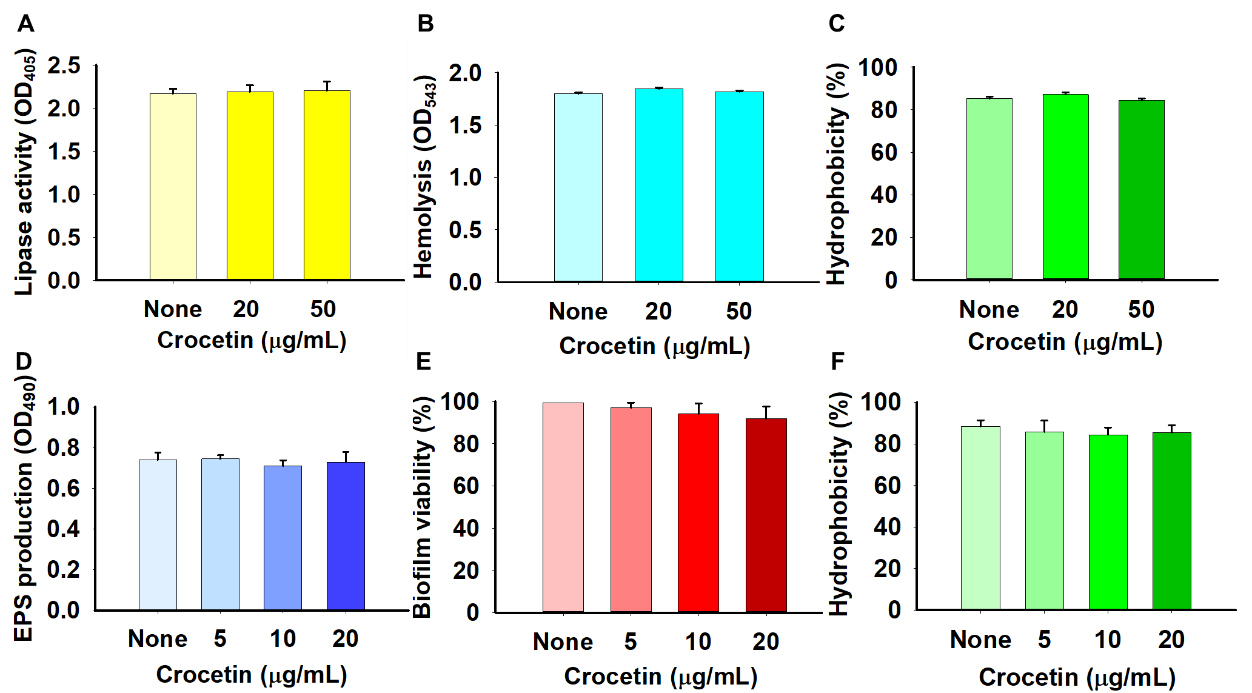


**Supplementary Figure 2.** Effects of crocetin on lipase activity (A), hemolytic activity (B), and hydrophobicity (C) of *S. aureus* ATCC 6538, and on exopolysaccharide production (D), biofilm viability (E), and hydrophobicity (F) of *S. epidermidis* ATCC 14990.


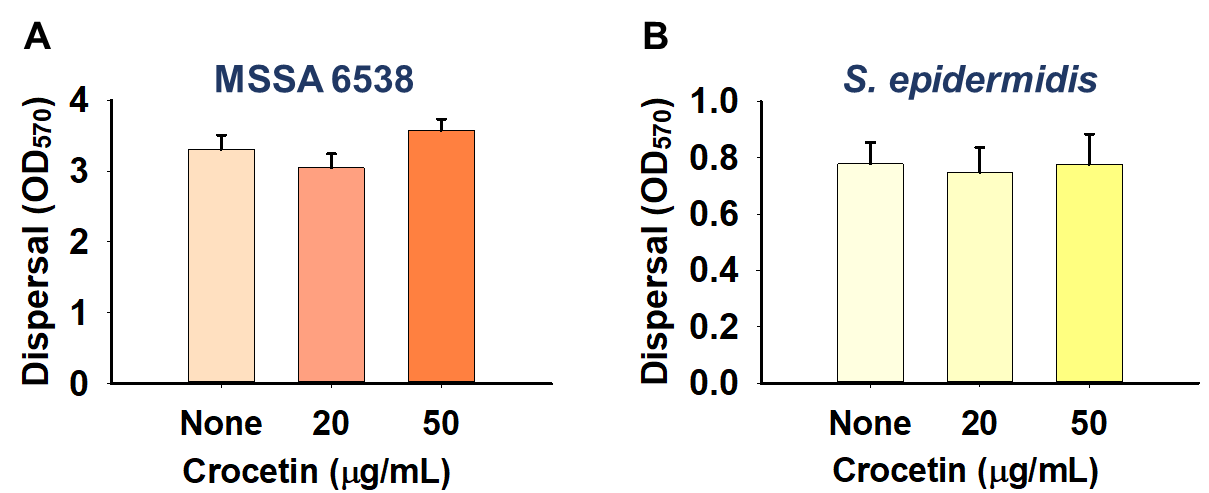


**Supplementary Figure 3.** Effects of crocetin on mature biofilms of *S. aureus* ATCC 6538 (A), and *S. epidermidis* ATCC 14990.
